# Too Much Walking

**Published:** 1886-06

**Authors:** 


**Too much walking.**—A great many physicians make a mistake in this thing, advising their patients to walk for exercise, when they would do them greater service by advising them to use the chest and arms for exercise. I know of nothing more insipid than walking for the sake of walking, or, in other words, taking set walks for the health. If united to this object, there is another; as, going to see a friend, going on errands of benevolence, searching for botanical or other specimens, or walking with pleasant company; then, if one's strength has not been previously overtaxed, a walk is beneficial. Milliners, dressmakers, book-keepers, and those who sit all day long, would be greatly profited in this way. But clerks, teachers, and those who feel exhausted after a day's work, may be still further wearied instead of refreshed, and all classes should contrive to find some way of calling the muscles of the upper extremities into action. The healthlift and some of the gymnastic exercises are good for this. The tendency of a great deal of exhaustive exercise is to draw the muscles and internal organs downward. This is why too much walking is objectionable. The womb especially tends to fall. Calling the arms and chest into action counteracts this tendency. It draws upward, and helps better than anything else to bring the womb to its place when it has prolapsed ; strengthens the ligaments so that it will remain in place, and prevent prolapsus if it has never occurred. No one should depend wholly upon walking for exercise, but alternate it with arm exercise.—*For Girls.*

